# Obesity: An Independent Predictor of Acute Renal Failure After General Surgery

**DOI:** 10.7759/cureus.71633

**Published:** 2024-10-16

**Authors:** Ananya Srivastava, Brodie Nolan, James J Jung

**Affiliations:** 1 Department of Anesthesiology and Pain Medicine, University of Toronto, Toronto, CAN; 2 Department of Emergency Medicine, St. Michael’s Hospital, Toronto, CAN; 3 Department of Surgery, Duke University, Durham, USA

**Keywords:** acute renal injury, obesity, obesity and abdominal surgeries, perioperative risk factors, postoperative outcomes

## Abstract

Background

Half of Americans will have obesity, and a quarter will have severe obesity by the year 2030. Postoperative acute renal failure (ARF) is associated with increased morbidity and mortality. Given the increase in the number of patients with obesity undergoing elective surgery, we investigated the relationship between obesity and postoperative ARF after elective general surgery procedures.

Methods

We performed a retrospective cohort study of patients in the 2015-2019 National Surgical Quality Improvement Program database who underwent elective general surgery procedures. The primary outcome was the presence of postoperative ARF. The patient body mass index (BMI) was categorized as normal (BMI 18.5-24.9), overweight (BMI 25-29.9), obesity class 1 and 2 (BMI 30-39.9), severe obesity (BMI 40-49.9), and extreme obesity (BMI³50). Descriptive statistics and unadjusted comparisons were performed for patients who developed postoperative ARF and those who did not. Multivariable regression analyses were used to model BMI categories and postoperative ARF, adjusting for patient- and surgical-level covariates.

Results

Among 424,527 patients included in the study, 3638 patients (0.8%) developed ARF. Patients who developed ARF were older, had a higher BMI, and had more serious comorbidities. After risk adjustment, there was a stepwise rise in odds of developing postoperative ARF with increasing BMI categories compared to normal BMI: (overweight: OR 1.11 (95% CI 1.0-1.23), obesity class 1 and 2: OR 1.32 (95% CI 1.2-1.46), severe obesity: OR 1.45 (95% CI 1.27-1.66), and extreme obesity: OR 1.78 (95% CI 1.47-2.15)).

Conclusion

Obesity is independently associated with ARF after elective general surgery procedures.

## Introduction

Over 40% of adults in the United States are diagnosed with obesity which is associated with serious comorbidities, such as diabetes mellitus, cardiovascular diseases, cancers, and renal diseases [1-3]. Body mass index (BMI) measures weight in relation to height and is often used to diagnose obesity. A normal BMI range is between 18.5-24.9 kg/m^2^, and an overweight BMI range is between 25-29.9 kg/m^2^. Obesity is defined as a BMI greater than or equal to 30 kg/m^2^. It can be further broken down into obesity class 1 (30-34.9 kg/m^2^), class 2 (35-39.9 kg/m^2^), and severe obesity (greater than or equal to 40 kg/m^2^). BMI equal to or greater than 50 kg/m^2^ is also known as extreme obesity. With the growing prevalence of obesity in the general population, there is an increasing number of patients with obesity who undergo elective surgery. Perioperatively, elevated BMI is associated with an increase in operating time, risk of infection, and length of hospitalization [4].

Acute renal failure (ARF) is defined as a rapid fall in the rate of glomerular filtration, which manifests clinically as an abrupt and sustained increase in the serum levels of urea and creatinine that may or may not require dialysis [5]. It is associated with poor clinical outcomes, such as increased length of hospital stay, subsequent development of chronic kidney disease, and increased in-hospital mortality [6,7]. Previous work has shown that elevated BMI is an independent risk factor for acute kidney injury in critically ill patients [8], and this has been reproduced in various postsurgical populations [9,10]. However, many of these studies were performed on small datasets. Further, there is a lack of empirical evidence on the relationship between elevated BMI and postoperative ARF following general surgery. Thus, the objective of this study was to determine the association between increasing BMI and the development of ARF within 30 days of an elective general surgery procedure using a large, multicenter dataset of surgical patients from across the United States and Canada.

## Materials and methods

Study design

The study was conducted at the Department of Surgery, University of Toronto, Toronto, Canada. We performed a retrospective cohort study of adult patients who underwent elective general surgery procedures and were accounted for in the American College of Surgeons National Surgical Quality Improvement Program® (ACS NSQIP®) database [11] to determine the relationship between BMI and postoperative ARF. Multivariable regression analyses were performed to determine the relationship between BMI categories and postoperative ARF after adjusting for pertinent patient- and surgical-level confounders. This study followed the Strengthening the Reporting of Observational Studies in Epidemiology (STROBE) reporting guidelines.

Data sources

The ACS NSQIP® Participant Use Data Files (PUF) from January 1, 2015, to December 31, 2019, were used for this study [11]. Trained and calibrated surgical clinical reviewers collect data from patient charts at each participating hospital using standardized definitions [12,13]. The PUFs contain aggregate, high-quality, standardized clinical data on preoperative, intraoperative, and postoperative variables from over 700 hospitals in the United States and Canada [13]. The PUFs do not identify hospitals, healthcare providers, or patients in compliance with the Health Insurance Portability and Accessibility Act (HIPAA). The Research Ethics Board at Unity Health (Toronto, ON) approved this study (REB ID 00042883).

Study population

All adult (age ≥18) patients who underwent elective general surgery operations as designated by the ACS NSQIP® were included in this study. Exclusion criteria included emergency surgery, outpatient surgery, presence of preoperative acute renal failure or preoperative use of dialysis, age above 80, and cases with missing data. Patients with a length of stay of less than two days were excluded due to the likelihood of a missed diagnosis of ARF during relatively short inpatient admissions.

Outcomes

The primary outcome of the study was the presence of postoperative ARF within 30 days after surgery. We used two variables in the NSQIP database to define this outcome: progressive renal insufficiency (using a variable named RENAINSF) and acute renal failure requiring dialysis (a variable named OPRENAFL). Progressive renal insufficiency is defined as a decreased kidney function as indicated by an increase in serum creatinine by >2 mg/dl from preoperative levels with no requirement for dialysis. Acute renal failure requiring dialysis is defined as the development of renal failure requiring hemodialysis, peritoneal dialysis, hemofiltration, hemodiafiltration, or ultrafiltration in a patient who did not require preoperative dialysis [13]. Secondary outcomes included 30-day readmission (using variable READMISSION), 30-day unplanned reoperation (using variable REOPERATION1), and 30-day all-cause mortality (using variable DOpertoD).

Covariates

Covariates considered in the multivariable analyses included patient characteristics such as BMI, sex, race (American Indian or Alaska Native, Asian, Black or African American, Native Hawaiian or Pacific Islander, White and Unknown or Not Reported), Hispanic ethnicity, age, and health-related variables such as smoking, dyspnea, functional health status, ventilator dependence, severe chronic obstructive pulmonary disease, ascites, congestive heart failure, hypertension requiring medication, disseminated cancer, immunosuppressant used for a chronic condition, greater than 10% loss of body weight in the six months before surgery, and bleeding disorders. Preoperative laboratory variables of serum creatinine and blood urea nitrogen were included. Perioperative variables, such as red blood cell (RBC) transfusion within 72 hours before surgery, wound classification, American Society of Anesthesiologists (ASA) classification, wound closure, sepsis present at the time of surgery, deep incisional surgical site infection (SSI) present at the time of surgery, organ/space SSI present at the time of surgery, pneumonia present at the time of surgery, on ventilator greater than 48 hours present at the time of surgery, sepsis within 48 hours before surgery, septic shock present at the time of surgery, principal anesthesia technique, quarter of admission, year of operation, work relative value unit, and total operation time were also considered in this study. Information about hospital and surgeon-specific variables is not reported in the NSQIP database and was therefore not included in the analysis.

Statistical analysis

Distributions of data points in continuous variables were described with mean (standard deviation, (SD)) or median (interquartile range (IQR)) for normal and non-normal distributions, respectively. Frequency analyses (%) were used to summarize data points in categorical variables. Several pre-specified associations were investigated between patient- and surgical-level variables and the outcomes. The accepted statistical practice of considering no more than one explanatory variable for every 10 patients who experienced an outcome was employed for variable selection [14]. Univariate logistic regression was used to investigate the relationship between the explanatory variables and postoperative ARF. All pairwise associations between each explanatory variable were studied to ensure none was overly correlated. BMI categories and explanatory variables with p≤0.05 on univariate analyses were entered into a multivariable logistic model using forward selection. The same processes were repeated to develop multivariable models predicting secondary outcomes. Goodness-of-fit was examined for all multivariable models using c-statistics and five-fold cross-validation with mean square error. Confidence intervals and p-values reported reflect a two-tailed α level of 0.05. Statistical analyses were conducted using R software version 3.6.3 (R Foundation for Statistical Computing, Vienna, Austria, https://www.R-project.org/)

## Results

Characteristics of study patients

**Figure 1:**
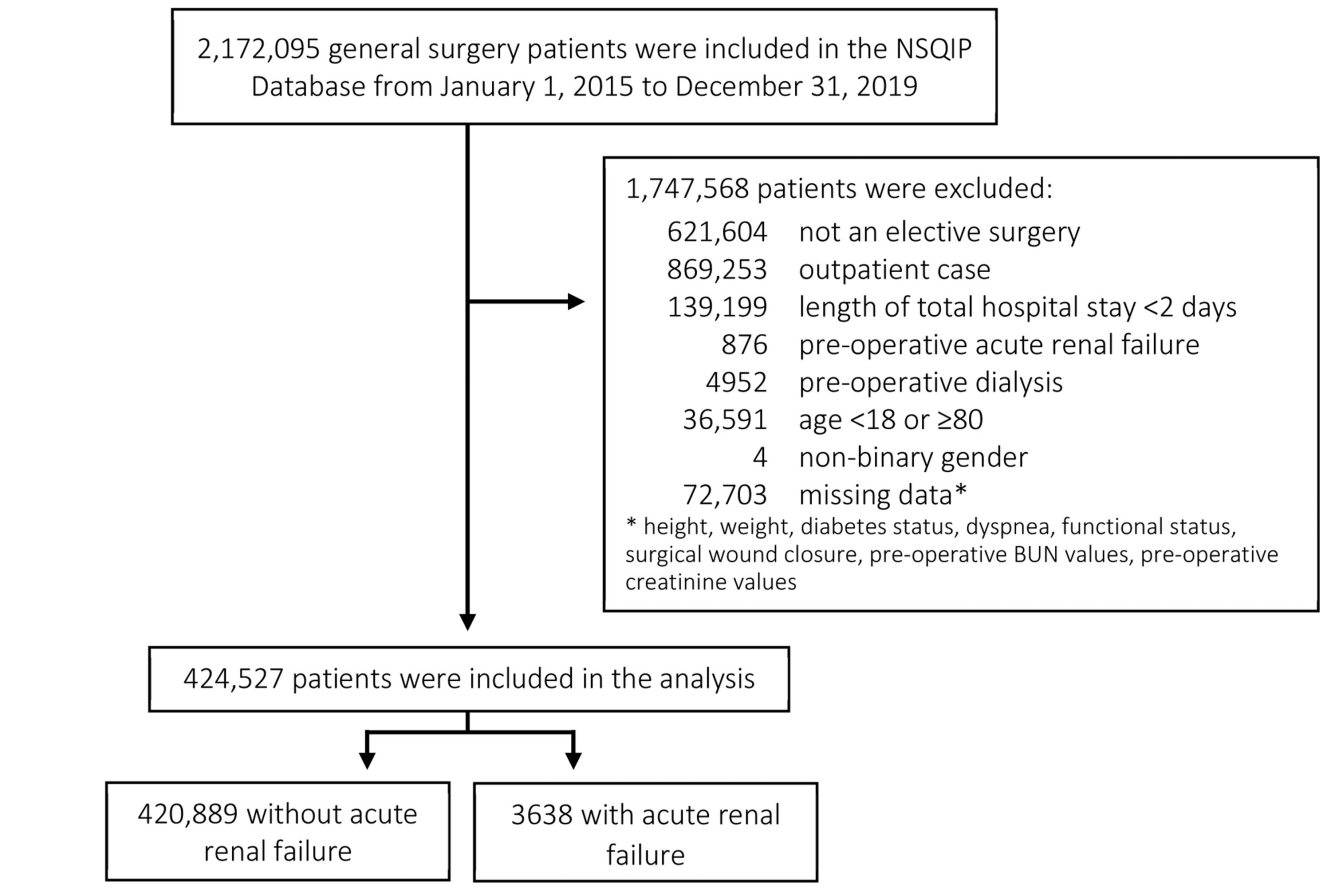
Study sample NSQIP: National Surgical Quality Improvement Program

Out of the 5,011,560 patients who were accounted for in the NSQIP database between January 1, 2015, and December 31, 2019, 2,172,095 patients underwent a general surgery procedure. After exclusion, a total of 424,527 patients were included in the final analysis, with 3638 patients who developed postoperative ARF within 30 days (Figure 1). Of the patients who developed ARF after surgery, 63.2% had progressive renal insufficiency, and 37.6% developed acute renal failure requiring dialysis.

Unadjusted comparisons between patients with ARF and patients without ARF

Patients with ARF were generally older (median age of 64 (IQR 56-71) years vs. 58 (IQR 48-67) years, p<0.001), less likely to be female (38.9% vs. 57.1%, p<0.001), and more likely to be a current smoker within one year (21.9% vs. 16.7%, p<0.001) than those without ARF (Table 1). Patients with ARF had a higher median BMI (30.9 (IQR 26.4-32.3) kg/m^2^ vs. 30.1 (IQR 25.8-31.4) kg/m^2^, p<0.001). Further, they were more likely to have diabetes (33.0% vs. 19.1%, p<0.001), severe chronic obstructive pulmonary disease (8.7% vs. 4.3%, p<0.001), bleeding disorders (5.4% vs. 2.5%, p<0.001), hypertension requiring medication (71.7% vs. 46.7%, p<0.001), congestive heart failure (2.3% vs. 0.6%, p<0.001), disseminated cancer (10.9% vs. 6.9%, p<0.001), recent significant weight loss (6.0% vs. 3.5%, p<0.001), and use of immunosuppressants (8.2% vs. 6.2%, p<0.001) (Table 1). Patients who developed ARF also had higher preoperative blood urea nitrogen (BUN) values (mean of 19.1 (SD 11.9) mg/dL vs. 15.2 (SD 7.5) mg/dL, p<0.001) and preoperative creatinine values (mean of 1.1 (SD 0.66) mg/dL vs. 0.9 (SD 0.4) mg/dL, p<0.001). Patients who developed ARF were more likely to have developed sepsis within 48 hours prior to surgery (2.8% vs. 1.4%, p<0.001), and more likely to have contaminated or dirty/infected wounds (20.3% vs. 14.0%, p<0.001) (Table 1). Patients who developed ARF were also more likely to have had either no layers surgically closed (1.2% vs. 0.4%, p<0.001) or only deep layers closed (1.7% vs. 1.0%, p<0.001) (Table 1). As well, patients who developed ARF generally had longer surgeries (mean of 255.3 (SD 147.8) minutes vs. 190.0 (SD 119.6) minutes, p<0.001) (Table 1). In unadjusted comparison analyses of postoperative outcomes, patients who developed ARF were also more likely to require a return to the operating room (30.7% vs. 4.0%, p<0.001) and had a longer length of stay in the hospital (mean 13.3 (SD 12.7) days vs. 5.2 (SD 5.0) days, p<0.001) (Table 2). As well, they were much more likely to require readmission to the hospital within 30 days (43.6% vs. 9.5%, p<0.001) (Table 2).

**Table 1:** Unadjusted comparison of baseline patient characteristics with and without postoperative acute renal failure BMI: body mass index; IQR: interquartile range; ASA: American Society of Anesthesiologists; SD: standard deviation; ARF: acute renal failure; RBC: red blood cell; SIRS: systemic inflammatory response syndrome * Chi-squared tests for comparisons of categorical variables and Mann-Whitney U tests for continuous variables. ** Work relative value unit: used to determine physician reimbursement under the Medicare Physician Fee Schedule by assessing the amount of time, skill, and training required to deliver a specific medical service.

Variable		No acute renal failure (n = 420889)	Acute renal failure (n = 3638)	p-value*
Body mass index (BMI), median (IQR)		29.6 (25.1-36.2)	30.4 (26.0-36.5)	<0.001
BMI category (%)	<18.5 kg/m^2^	8086 (1.9)	59 (1.6)	<0.001
	18.5-24.9 kg/m^2^	94663 (22.5)	665 (18.3)	
	25-29.9 kg/m^2^	116970 (27.8)	1025 (28.2)	
	30-39.9 kg/m^2^	132303 (31.4)	1331 (36.6)	
	40-49.9 kg/m^2^	51296 (12.2)	403 (11.1)	
	>50 kg/m^2^	17571 (4.2)	155 (4.3)	
Sex (%)	Female	240508 (57.1)	1417 (38.9)	<0.001
Race (%)	American Indian or Alaska Native	1973 (0.5)	25 (0.7)	<0.001
	Asian	13241 (3.1)	84 (2.3)	
	Black or African American	45362 (10.8)	579 (15.9)	
	Native Hawaiian or Pacific Islander	1153 (0.3)	11 (0.3)	
	White	310645 (73.8)	2559 (70.3)	
	Unknown/Not Reported	48515 (11.5)	380 (10.4)	
Hispanic ethnicity (%)	Yes	31594 (7.5)	242 (6.7)	0.138
	No	350307 (83.2)	3063 (84.2)	
	Unknown	38988 (9.3)	333 (9.2)	
Age, median (IQR)		58 (48-67)	64 (56-71)	<0.001
Diabetes mellitus requiring therapy (%)	Insulin therapy	29161 (6.9)	536 (14.7)	<0.001
	Non-insulin therapy	51059 (12.1)	663 (18.2)	
	No	340669 (80.9)	2439 (67.0)	
Current smoker within one year (%)		70488 (16.7)	795 (21.9)	<0.001
Dyspnea (%)	At rest	1407 (0.3)	31 (0.9)	<0.001
	Moderate exertion	28025 (6.7)	419 (11.5)	
	No	391457 (93.0)	3188 (87.6)	
Functional health status (%)	Totally dependent	929 (0.2)	9 (0.2)	
	Partially dependent	5010 (1.2)	90 (2.5)	<0.001
	Independent	414950 (98.6)	3539 (97.3)	
Ventilator dependent (%)		131 (0.0)	5 (0.1)	0.002
History of severe chronic obstructive pulmonary disease (%)		18197 (4.3)	316 (8.7)	<0.001
Ascites <30 days prior to surgery (%)		1057 (0.3)	46 (1.3)	<0.001
Congestive heart failure <30 days prior to surgery (%)		2323 (0.6)	84 (2.3)	<0.001
Hypertension requiring medication (%)		196453 (46.7)	2610 (71.7)	<0.001
Disseminated cancer (%)		28854 (6.9)	396 (10.9)	<0.001
Immunosuppressant used for a chronic condition (%)		25917 (6.2)	298 (8.2)	<0.001
> 10% weight loss in the 6 months prior to surgery (%)		14824 (3.5)	220 (6.0)	<0.001
Bleeding disorders (%)		10707 (2.5)	195 (5.4)	<0.001
RBC transfusion <72 hours prior to surgery		1364 (0.3)	25 (0.7)	<0.001
Pre-operative blood urea nitrogen value, mean ± SD		15.20 ± 7.46	19.14 ± 11.90	<0.001
Pre-operative creatinine value, mean ± SD		0.89 ± 0.39	1.14 ± 0.66	<0.001
Wound classification (%)	1-Clean	68214 (16.2)	427 (11.7)	<0.001
	2-Clean/Contaminated	293812 (69.8)	2473 (68.0)	
	3-Contaminated	40021 (9.5)	484 (13.3)	
	4-Dirty/Infected	18842 (4.5)	254 (7.0)	
ASA classification (%)	1-No disturb	7021 (1.7)	14 (0.4)	<0.001
	2-Mild disturb	160679 (38.2)	704 (19.4)	
	3-Severe disturb	237495 (56.4)	2565 (70.5)	
	4-Life threat	15173 (3.6)	353 (9.7)	
	5-Moribund	37 (0.0)	1 (0.0)	
	None assigned	484 (0.1)	1 (0.0)	
Surgical wound closure (%)	All layers fully closed	414647 (98.5)	3533 (97.1)	<0.001
	No layers surgically closed	1852 (0.4)	42 (1.2)	
	Only deep layers closed	4390 (1.0)	63 (1.7)	
Sepsis within 48 hours prior to surgery (%)	Septic shock	92 (0.0)	10 (0.3)	<0.001
	Sepsis	1779 (0.4)	32 (0.9)	
	SIRS	4142 (1.0)	60 (1.6)	
	None	414876 (98.6)	3536 (97.2)	
Present at the time of surgery (%)	Sepsis	2277 (0.5)	42 (1.2)	<0.001
	Septic shock	182 (0.0)	30 (0.8)	<0.001
	Deep incisional SSI	2477 (0.6)	80 (2.2)	0.008
	Organ/Space SSI	310 (0.1)	16 (0.4)	<0.001
	Pneumonia	78 (0.0)	13 (0.4)	<0.001
	On ventilator > 48h	405 (0.1)	9 (0.2)	<0.001
Quarter of admission (%)	1	108498 (25.8)	978 (26.9)	0.486
	2	104374 (24.8)	898 (24.7)	
	3	103081 (24.5)	874 (24.0)	
	4	104936 (24.9)	888 (24.4)	
Principal anesthesia technique (%)	Epidural	430 (0.1)	7 (0.2)	0.551
	General	418333 (99.4)	3617 (99.4)	
	Local	48 (0.0)	0 (0.0)	
	MAC/IV Sedation	704 (0.2)	6 (0.2)	
	None	90 (0.0)	0 (0.0)	
	Other	350 (0.1)	2 (0.1)	
	Regional	206 (0.0)	1 (0.0)	
	Spinal	692 (0.2)	4 (0.1)	
	Unknown	36 (0.0)	1 (0.0)	
Work relative value unit**, mean ± SD		25.31 ± 11.51	29.12 ± 12.82	<0.001
Total operation time in minutes, mean ± SD		190.03 ± 119.64	255.28 ± 147.83	<0.001

**Table 2:** Unadjusted postoperative comparisons of patients with acute renal failure and without acute renal failure ARF: acute renal failure; SSI: surgical site infection; CPR: cardio-pulmonary resuscitation; RBC: red blood cell; SD: standard deviation * Chi-squared tests for comparisons of categorical variables and Mann-Whitney U tests for continuous variables.

Variable		No acute renal failure n = 420889	Acute renal failure n = 3638	p-value*
Superficial incisional SSI (%)		13587 (3.2)	246 (6.8)	<0.001
Deep incisional SSI (%)		3193 (0.8)	77 (2.1)	<0.001
Organ/Space SSI (%)		17797 (4.2)	853 (23.4)	<0.001
Wound disruption (%)		2916 (0.7)	103 (2.8)	<0.001
Pneumonia (%)		6214 (1.5)	674 (18.5)	<0.001
Unplanned intubation (%)		3753 (0.9)	836 (23.0)	<0.001
Pulmonary embolism (%)		2209 (0.5)	85 (2.3)	<0.001
On ventilator greater than 48 hours (%)		2769 (0.7)	842 (23.1)	<0.001
Progressive renal insufficiency (%)		0 (0.0)	2298 (63.2)	<0.001
Acute renal failure (%)		0 (0)	1368 (37.6)	<0.001
Urinary tract infection (%)		6687 (1.6)	230 (6.3)	<0.001
Stroke/Cerebrovascular accident (%)		526 (0.1)	35 (1.0)	<0.001
Cardiac arrest requiring CPR (%)		1061 (0.3)	300 (8.2)	<0.001
Myocardial infarction (%)		1521 (0.4)	181 (5)	<0.001
RBC within the first 72h of surgery start time (%)		25280 (6.0)	860 (23.6)	<0.001
Deep vein thrombosis/thrombophlebitis (%)		3699 (0.9)	198 (5.4)	<0.001
Sepsis (%)		10988 (2.6)	455 (12.5)	<0.001
Septic shock (%)		2765 (0.7)	884 (24.3)	<0.001
30 day reoperation (%)		17036 (4.0)	1117 (30.7)	<0.001
Still in hospital > 30 days (%)		1328 (0.3)	218 (6.0)	<0.001
	No	380840 (90.5)	2053 (56.4)	<0.001
30-day readmission	Unknown	28 (0.0)	0 (0.0)	
	Yes	40021 (9.5)	1585 (43.6)	
Days from operation to discharge (mean ± SD)		5.05 ± 4.67	13.01 ± 12.38	<0.001
Length of total hospital stay in days (mean ± SD)		5.24 ± 5.03	13.30 ± 12.66	<0.001

Multivariable models predicting ARF 

**Figure 2:**
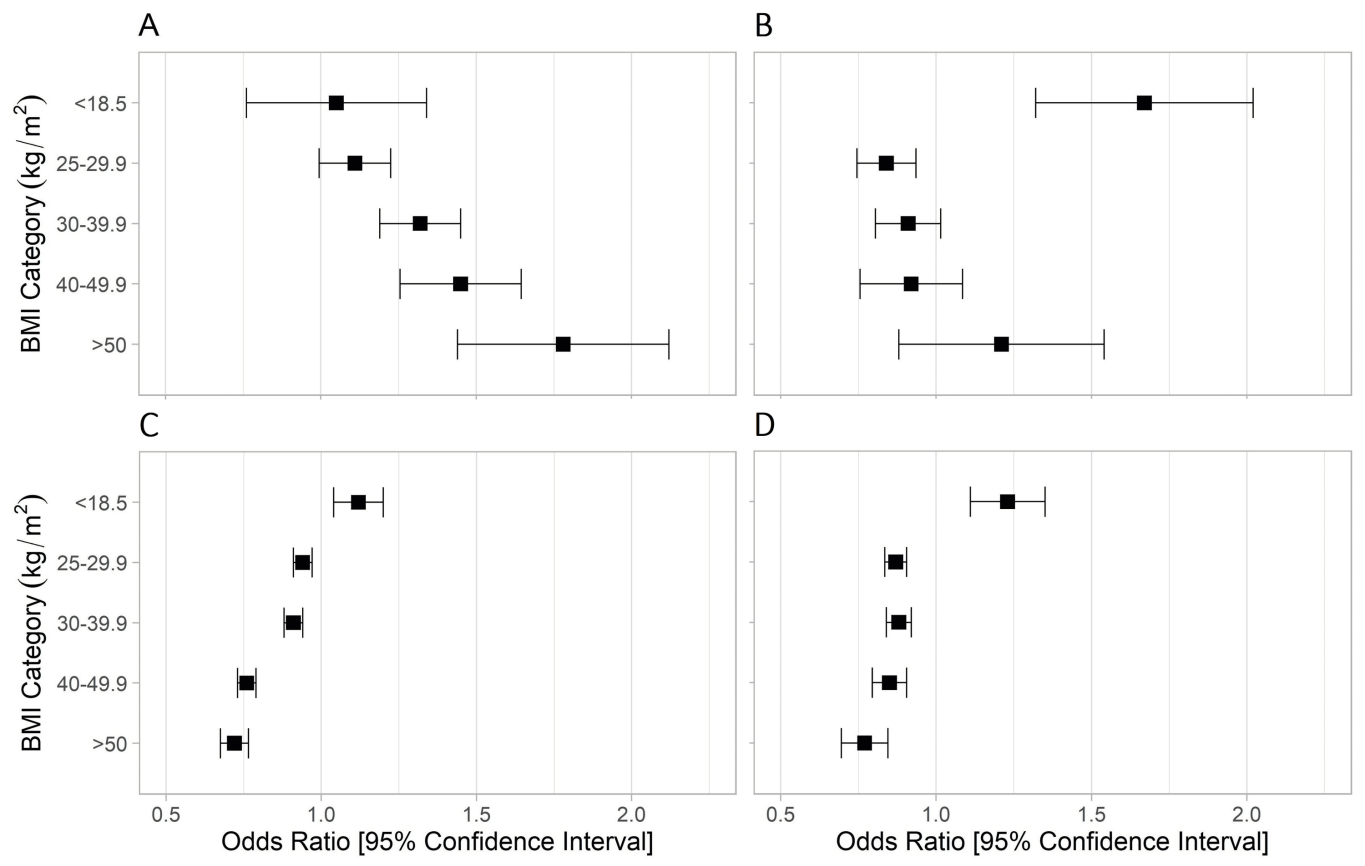
Multivariable regression analyses to assess the relationship between BMI categories and outcomes. Multivariable regression analyses to assess the relationship between BMI categories and the following postoperative outcomes: A) acute renal failure; B) mortality; C) readmission; and D) reoperation. Normal BMI (18.5-24.9 kg/m2) was used as the reference. Odds ratios and 95% confidence intervals are shown on the x-axis, with BMI categories on the y-axis. BMI: body mass index

Multivariable regression analyses demonstrated that there was a stepwise increase in odds of postoperative ARF with increasing BMI categories compared to normal BMI category (overweight: OR 1.11 (95% CI 1.0-1.23), class 1 and 2 obesity: OR 1.32 (95%-CI 1.2-1.46), severe obesity: OR 1.45 (95%-CI 1.27-1.66), and extreme obesity: OR 1.78 (95%-CI 1.47-2.15)) (Figure 2). There was no difference in the odds of postoperative ARF in the underweight BMI compared to the normal BMI. Other independent predictors of postoperative ARF included male sex (OR 1.60 (95%-CI 1.49-1.72)), increasing age (OR 1.02 (95%-CI 1.02-1.02)), current smoker (OR 1.46 (95%-CI 1.34-1.59)), hypertension requiring medication (OR 1.75 (95%-CI 1.61-1.90)), ascites (OR 3.48 (95%-CI 2.55-4.77)), and immunosuppressive use (OR 1.44 (95%-CI 1.28-1.63)) (Table 3).

**Table 3:** Multivariable logistic regression model predicting postoperative acute renal failure BMI: body mass index; OR: odds ratio; CI: confidence interval; ARF: acute renal failure; RBC: red blood cell; ASA: American Society of Anesthesiologists; SSI: surgical site infection; SIRS: systemic inflammatory response syndrome

Characteristic		OR	95% CI	p-value
BMI category	18.5-25 kg/m^2^	ref	-	-
	<18.5 kg/m^2^	1.05	0.80-1.38	0.719
	25-29.9 kg/m^2^	1.11	1.00-1.23	0.045
	30-39.9 kg/m^2^	1.32	1.20-1.46	<0.001
	40-49.9 kg/m^2^	1.45	1.27-1.66	<0.001
	>50 kg/m^2^	1.78	1.47-2.15	<0.001
Sex	Female	ref	-	-
	Male	1.60	1.49-1.72	<0.001
Race	American Indian or Alaska Native	ref	-	-
	Asian	0.59	0.37-0.93	0.022
	Black or African American	1.05	0.70-1.58	0.815
	Native Hawaiian or Pacific Islander	0.84	0.41-1.72	0.625
	Unknown/Not Reported	0.63	0.41-0.96	0.031
	White	0.66	0.44-0.98	0.040
Hispanic ethnicity	No	ref	-	-
	Unknown	1.18	1.01-1.38	0.040
	Yes	1.20	1.04-1.38	0.011
Age		1.02	1.02-1.02	<0.001
Diabetes mellitus requiring therapy	Insulin	ref	-	-
	No	0.78	0.71-0.87	<0.001
	Non-insulin	0.93	0.83-1.05	0.232
Current smoker within one year	No	ref	-	-
	Yes	1.46	1.34-1.59	<0.001
Dyspnea	At rest	ref	-	-
	Moderate exertion	0.89	0.61-1.30	0.542
	No	0.71	0.49-1.04	0.075
Functional health status	Independent	ref	-	-
	Partially dependent	1.22	0.98-1.53	0.075
	Totally dependent	0.77	0.39-1.51	0.445
Ventilator dependent	No	ref	-	-
	Yes	0.49	0.15-1.56	0.229
History of severe chronic obstructive pulmonary disease	No	ref	-	-
	Yes	1.20	1.05-1.36	0.006
Ascites <30 days prior to surgery	No	ref	-	-
	Yes	3.48	2.55-4.77	<0.001
Congestive heart failure <30 days prior to surgery	No	ref	-	-
	Yes	1.59	1.26-2.01	<0.001
Hypertension requiring medication	No	ref	-	-
	Yes	1.75	1.61-1.90	<0.001
Disseminated cancer	No	ref	-	-
	Yes	1.37	1.23-1.53	<0.001
Immunosuppressant used for a chronic condition	No	ref	-	-
	Yes	1.44	1.28-1.63	<0.001
>10% weight loss in the 6 months prior to surgery	No	ref	-	-
	Yes	1.16	1.00-1.34	0.045
Bleeding disorders	No	ref	-	-
	Yes	1.35	1.16-1.57	<0.001
RBC transfusion <72 hours prior to surgery	No	ref	-	-
	Yes	0.96	0.63-1.48	0.865
Pre-operative blood urea nitrogen value		1.02	1.01-1.02	<0.001
Pre-operative creatinine value		1.33	1.28-1.37	<0.001
Wound classification	1 - Clean	ref	-	-
	2 - Clean/Contaminated	1.24	1.11-1.38	<0.001
	3 - Contaminated	1.62	1.42-1.86	<0.001
	4 - Dirty/Infected	1.56	1.31-1.85	<0.001
ASA classification	1 - No Disturb	ref	-	-
	2 - Mild Disturb	1.24	0.73-2.12	0.422
	3 - Severe Disturb	1.75	1.03-2.98	0.038
	4 - Life Threat	2.24	1.30-3.86	0.004
	5 - Moribund	0.47	0.04-5.33	0.540
	None assigned	0.42	0.06-3.24	0.408
Wound closure	All layers fully closed	ref	-	-
	No layers surgically closed	1.79	1.28-2.49	0.001
	Only deep layers closed	1.41	1.08-1.83	0.011
Sepsis within 48 hours prior to surgery	None	ref	-	-
	Sepsis	1.22	0.82-1.82	0.334
	Septic shock	1.20	0.47-3.03	0.702
	SIRS	1.52	1.17-1.98	0.002
Sepsis present at time of surgery	No	ref	-	-
	Yes	0.91	0.65-1.28	0.603
Septic shock present at time of surgery	No	ref	-	-
	Yes	4.05	2.47-6.65	<0.001
Deep incisional SSI present at the time of surgery	No	ref	-	-
	Yes	1.40	0.70-2.79	0.342
Organ/Space SSI present at the time of surgery	No	ref	-	-
	Yes	2.11	1.64-2.72	<0.001
Pneumonia present at the time of surgery	No	ref	-	-
	Yes	2.64	1.53-4.57	<0.001
On ventilator > 48 hours present at the time of surgery	No	ref	-	-
	Yes	6.28	2.85-13.86	<0.001
Work relative value unit		1.01	1.00-1.01	<0.001
Total operation time		1.00	1.00-1.00	<0.001

Underweight BMI was independently associated with increased odds of death (OR 1.67 (95% CI 1.36-2.06)) (Tables 4, 5, Appendices 1, 2), and overweight BMI was associated with decreased odds of death (OR 0.84 (95% CI 0.75-0.94)). Obesity was not associated with postoperative death compared to the normal BMI. Underweight BMI was independently associated with an increased risk of readmission (OR 1.12 (95% CI 1.04-1.20)) (Tables 6, 7, Appendices 3, 4). Increasing BMI compared to normal BMI was associated with lower odds of readmission (overweight: OR 0.94 (95% CI 0.91-0.97), class 1 and 2 obesity: OR 0.91 (95% CI 0.88-0.94), severe obesity: OR 0.76 (95% CI 0.73-0.79), and extreme obesity: OR 0.72 (95% CI 0.68-0.77)) (Tables 6, 7). Similarly, underweight BMI was independently associated with higher odds of reoperation (OR 1.23 (95% CI 1.12-1.36)) and increasing BMI was associated with lower odds of reoperation (overweight: OR 0.87 (95% CI 0.83-0.90), class 1 and 2 obesity: OR 0.88 (95% CI 0.84-0.92), severe obesity: OR 0.85 (95% CI 0.80-0.91), and extreme obesity: OR 0.77 (95% CI 0.70-0.85)) (Tables 8, 9, Appendices 5, 6).

Model validation

The calculated mean square error values and c-statistics from five-fold cross-validation on all our multivariable regression models demonstrated model consistency. Mean square error and c-statistic values are reported in Appendix 7.

## Discussion

We performed a retrospective cohort study of 424,527 patients in the 2015-2019 NSQIP database who underwent elective general surgery procedures to demonstrate that increasing BMI was independently associated with postoperative ARF compared to normal BMI. We also showed that underweight BMI was associated with increased odds of postoperative death, readmission, and reoperation, while increasing BMI was associated with lower odds of readmission and reoperation. Our study represents one of the largest contemporary analyses to determine the relationship between BMI and postoperative ARF after elective general surgery.

Our study found a stepwise increase in the odds of developing postoperative ARF with increasing BMI in patients who underwent general surgery procedures. This finding is in line with previous work that found that elevated BMI was associated with increased risk of postoperative ARF in several types of surgical procedures, such as cardiac surgery [15,16], tracheostomies [17], pancreaticoduodenectomies [18], surgery for diverticular disease [19,20], and hip fractures [21]. Our study conducted robust risk-adjusted analyses on the relationship between BMI and postoperative ARF using a contemporary, multicenter, and multiyear database and focused on general surgery. Thus, the findings from our study add to the growing body of evidence linking obesity to postoperative ARF. The underlying pathophysiology behind the relationship between obesity and ARF is thought to involve multiple mechanisms, including chronic inflammation, oxidative stress, and endothelial dysfunction [10,22]. Obesity often leads to dyslipidemia, and the resultant lipid accumulation in the kidneys can promote inflammation, oxidative stress, and fibrosis, contributing to renal dysfunction [23]. Adipose tissue also releases pro-inflammatory cytokines and chemokines, leading to chronic low-grade inflammation and oxidative stress [24]. These harmful effects can contribute to renal damage. Elevated BMI can raise the glomerular filtration rate due to increased metabolic demand and renal blood flow, which can contribute to kidney damage over time [22]. Obesity is also linked to the activation of the renin-angiotensin-aldosterone system, resulting in increased renal vascular resistance, salt retention, and hypertension, all of which can contribute to kidney injury [22].

The relationship between underweight BMI and mortality has been studied in the past and demonstrated an increased risk of death among those with lower than normal BMI [25,26]. Our finding of increased morbidity and mortality for underweight patients is in line with the scientific literature. This supports the theory that most of the patients with underweight BMI are undernourished and more likely to have weakened immune systems, dietary deficits, and decreased physiologic reserve. Consequently, they are more likely to experience postoperative deaths, readmissions, and reoperations. A previous literature review indicated that underweight patients had higher crude mortality following coronary artery bypass graft surgery, indicating that undernutrition should be investigated further as a risk factor [27]. The low fat-free mass index was also linked to an increased likelihood of negative outcomes following cardiac surgery, stressing the importance of optimizing nutritional status for undernourished patients [28]. Monitoring the health of underweight patients is critical, as is providing adequate nutritional assistance to help mitigate this risk.

Our finding that increasing BMI was protective for readmission and reoperation has been previously described in studies and is known as the “obesity paradox” [29]. The obesity paradox challenges the paradigm of obesity as a perioperative risk factor. Although obesity is associated with various comorbidities, in patients who undergo acute stress, such as surgery and critical illness, increased BMI has been found to be a protective factor linked to lower morbidity and mortality [29]. This obesity paradox has been demonstrated in various study samples [29,30]. In critically ill patients, obesity has been linked to increased morbidity but not mortality, with one study showing that obesity was protective against mortality in these individuals. Our findings emphasize the complex relationship of obesity with health outcomes. More work is needed to fully understand the mechanisms underlying the obesity paradox and to determine the circumstances under which obesity may confer a survival benefit.

Decreasing the risk of postoperative ARF in patients with elevated BMI requires a comprehensive approach that targets the underlying processes and risk factors for both conditions. Losing weight through lifestyle modifications such as dietary changes and increased physical exercise has shown promise in improving renal function. In addition, our study identified diabetes requiring medication as a protective factor against ARF. This may be attributed to the effective management of diabetes and monitoring of renal function in these patients, which could mitigate the detrimental effects of obesity. This finding further supports the theory that managing comorbid chronic conditions such as hypertension and diabetes with appropriate medication and monitoring might help reduce the risk of ARF. Further, there may be an important role in educating patients with obesity on their increased risk of renal complications when they undergo general surgery procedures. Close monitoring of renal function in these patients is critical to identify and manage modifiable risk factors early. As well, patients may benefit from a multidisciplinary approach with involvement from physicians, nurses, dietitians, and fitness experts. Further consideration should be given to renal-sparing strategies, including careful drug choices and intraoperative fluid resuscitation.

There are limitations in this study. First, hospital-specific variables, such as geographic location or institution type, and surgeon-specific variables were not included in the analysis as the NSQIP does not report these variables. Second, despite adjusting for key confounders, the possibility of residual confounding remains, particularly from unmeasured factors such as lifestyle, socioeconomic status, or detailed comorbidity history, which could influence the observed associations. Third, we were not able to analyze outcomes that occurred beyond the 30 days after surgery because they were not reported in the NSQIP database. As a result, any delayed or chronic renal dysfunction may not have been captured. Fourth, while BMI was used as the primary measure of obesity, it does not capture other important aspects such as fat distribution or muscle mass, which may provide additional insights into the relationship between obesity and ARF. Lastly, patients with missing values for diabetes status, dyspnea, functional status, surgical wound closure, preoperative BUN values, and preoperative creatinine values were excluded; however, we were unable to probe further into why some patients did not have these values collected.

## Conclusions

Our study demonstrated that obesity was independently associated with increasing odds of developing ARF after elective general surgery procedures compared to the normal BMI. These findings emphasize the need for targeted interventions such as weight management, renal monitoring, and optimized perioperative care to reduce the incidence of ARF and improve outcomes in this population.

## Appendices

Appendix 1 

**Table 4:** NSQIP 2015-2019 univariable analysis of mortality BMI: body mass index; RBC: red blood cell; SSI: surgical site infection; MAC: monitored anesthesia care; ASA: American Society of Anesthesiologists; IV: intravenous; SIRS: systemic inflammatory response syndrome; NSQIP: National Surgical Quality Improvement Program

Characteristic		OR	95% CI	p-value
BMI category	18.5-25 kg/m^2^	REF		
	<18.5 kg/m^2^	1.98	1.63-2.42	<0.001
	25-29.9 kg/m^2^	0.83	0.75-0.92	<0.001
	30-39.9 kg/m^2^	0.76	0.69-0.85	<0.001
	40-49.9 kg/m^2^	0.47	0.40-0.56	<0.001
	>50 kg/m^2^	0.54	0.43-0.70	<0.001
BMI		0.97	0.97-0.98	<0.001
Sex	Female	0.00	0.00-0.00	<0.001
	Male	1.98	1.83-2.15	<0.001
Race	American Indian or Alaska Native	0.01	0.01-0.01	<0.001
	Asian	0.68	0.40-1.15	0.148
	Black or African American	0.71	0.43-1.16	0.167
	Native Hawaiian or Pacific Islander	0.60	0.24-1.54	0.289
	Unknown/Not Reported	0.64	0.39-1.04	0.073
	White	0.70	0.43-1.12	0.138
Hispanic ethnicity	No	0.01	0.01-0.01	<0.001
	Unknown	1.00	0.87-1.14	0.950
	Yes	0.71	0.59-0.84	<0.001
Age		1.07	1.07-1.08	<0.001
Diabetes mellitus requiring therapy	Insulin	0.01	0.01-0.01	<0.001
	No	0.49	0.44-0.56	<0.001
	Non-insulin	0.75	0.65-0.87	<0.001
Current smoker within one year	No	0.01	0.01-0.01	<0.001
	Yes	1.38	1.26-1.52	<0.001
Dyspnea	At rest	0.04	0.03-0.05	<0.001
	Moderate exertion	0.34	0.25-0.46	<0.001
	No	0.14	0.11-0.19	<0.001
Functional health status	Independent	0.01	0.01-0.01	<0.001
	Partially dependent	5.71	4.84-6.73	<0.001
	Totally dependent	9.11	6.74-12.33	<0.001
Ventilator dependent	No	0.01	0.01-0.01	<0.001
	Yes	16.40	9.05-29.69	<0.001
History of severe chronic obstructive pulmonary disease	No	0.01	0.01-0.01	<0.001
	Yes	3.36	2.99-3.78	<0.001
Ascites <30 days prior to surgery	No	0.01	0.01-0.01	<0.001
	Yes	11.36	8.86-14.57	<0.001
Congestive heart failure <30 days prior to surgery	No	0.01	0.01-0.01	<0.001
	Yes	6.60	5.31-8.19	<0.001
Hypertension requiring medication	No	0.00	0.00-0.00	<0.001
	Yes	2.18	2.01-2.37	<0.001
Disseminated cancer	No	0.01	0.00-0.01	<0.001
	Yes	3.33	3.01-3.68	<0.001
Immunosuppressant used for a chronic condition	No	0.01	0.01-0.01	<0.001
	Yes	1.34	1.16-1.55	<0.001
>10% weight loss in the 6 months prior to surgery	No	0.01	0.01-0.01	<0.001
	Yes	3.84	3.40-4.33	<0.001
Bleeding disorders	No	0.01	0.01-0.01	<0.001
	Yes	2.85	2.44-3.33	<0.001
RBC transfusion <72 hours prior to surgery	No	0.01	0.01-0.01	<0.001
	Yes	6.41	4.82-8.52	<0.001
Pre-operative blood urea nitrogen value		1.03	1.02-1.03	<0.001
Pre-operative creatinine value		1.30	1.25-1.35	<0.001
Wound classification	1 - Clean	0.01	0.00-0.01	<0.001
	2 - Clean/Contaminated	1.13	1.01-1.27	0.040
	3 - Contaminated	1.56	1.34-1.82	<0.001
	4 - Dirty/Infected	1.81	1.51-2.18	<0.001
ASA classification	1 - No disturb	0.00	0.00-0.00	<0.001
	2 - Mild disturb	2.71	1.12-6.57	0.027
	3 - Severe disturb	10.30	4.28-24.76	<0.001
	4 - Life threat	40.62	16.83-98.08	<0.001
	5 - Moribund	78.11	14.68-415.73	<0.001
	None assigned	8.75	2.09-36.71	0.003
Wound closure	All layers fully closed	0.01	0.01-0.01	<0.001
	No layers surgically closed	7.01	5.53-8.89	<0.001
	Only deep layers closed	2.07	1.57-2.72	<0.001
Sepsis within 48 hours prior to surgery	None	0.01	0.01-0.01	<0.001
	Sepsis	5.45	4.15-7.17	<0.001
	Septic shock	35.49	21.05-59.83	<0.001
	SIRS	3.71	2.98-4.61	<0.001
Sepsis present at the time of surgery	No	0.01	0.01-0.01	<0.001
	Yes	2.53	1.80-3.56	<0.001
Septic shock present at the time of surgery	No	0.01	0.01-0.01	<0.001
	Yes	37.39	26.26-53.23	<0.001
Deep incisional SSI present at the time of surgery	No	0.01	0.01-0.01	<0.001
	Yes	2.91	1.38-6.14	0.005
Organ/Space SSI present at the time of surgery	No	0.01	0.01-0.01	<0.001
	Yes	3.13	2.33-4.20	<0.001
Pneumonia present at the time of surgery	No	0.01	0.01-0.01	<0.001
	Yes	16.02	10.85-23.64	<0.001
On ventilator > 48 hours present at the time of surgery	No	0.01	0.01-0.01	<0.001
	Yes	54.25	33.52-87.80	<0.001
Principal anesthesia technique	Epidural	0.00	0.00-0.02	<0.001
	General	1.28	0.32-5.14	0.726
	Local	4.63	0.41-52.00	0.215
	MAC/IV Sedation	4.69	1.07-20.63	0.041
	None	0.00	0.00-0.00	0.947
	Other	3.13	0.60-16.25	0.174
	Regional	0.00	0.00-0.00	0.920
	Spinal	2.21	0.46-10.69	0.324
	Unknown	0.00	0.00-0.00	0.966
Quarter of admission	1	0.01	0.01-0.01	<0.001
	2	1.08	0.97-1.21	0.161
	3	0.98	0.88-1.10	0.747
	4	1.00	0.90-1.12	0.973
Work relative value unit		1.03	1.02-1.03	<0.001
Total operation time		1.00	1.00-1.00	<0.001

Appendix 2

**Table 5:** NSQIP 2015-2019 multivariable analysis of mortality BMI: body mass index; RBC: red blood cell; OD: odds ratio; MAC: monitored anesthesia care; IV: intravenous; CI: confidence interval; ASA: American Society of Anesthesiologists; SSI: surgical site infection; SIRS: systemic inflammatory response syndrome; NSQIP: National Surgical Quality Improvement Program

Characteristic		OR	95% CI	p-value
BMI category	18.5-25 kg/m^2^	ref	-	-
	<18.5 kg/m^2^	1.67	1.36-2.06	<0.001
	25-29.9 kg/m^2^	0.84	0.75-0.94	0.002
	30-39.9 kg/m^2^	0.91	0.81-1.02	0.089
	40-49.9 kg/m^2^	0.92	0.77-1.10	0.361
	>50 kg/m^2^	1.21	0.93-1.59	0.153
Sex	Female	ref	-	-
	Male	1.54	1.42-1.68	<0.001
Race	American Indian or Alaska Native	ref	-	-
	Asian	0.69	0.40-1.18	0.176
	Black or African American	0.80	0.49-1.33	0.395
	Native Hawaiian or Pacific Islander	0.75	0.29-1.94	0.558
	Unknown/Not Reported	0.64	0.38-1.07	0.091
	White	0.68	0.42-1.10	0.118
Hispanic ethnicity	No	ref	-	-
	Unknown	1.05	0.86-1.28	0.638
	Yes	1.08	0.89-1.30	0.429
Age		1.06	1.05-1.06	<0.001
Diabetes mellitus requiring therapy	Insulin	ref	-	-
	No	0.97	0.85-1.10	0.642
	Non-insulin	1.02	0.87-1.19	0.811
Current smoker within one year	No	ref	-	-
	Yes	1.30	1.17-1.44	<0.001
Dyspnea	At rest	ref	-	-
	Moderate exertion	0.72	0.52-1.00	0.049
	No	0.52	0.38-0.72	<0.001
Functional health status	Independent	ref	-	-
	Partially dependent	2.38	1.99-2.85	<0.001
	Totally dependent	4.28	3.03-6.05	<0.001
Ventilator dependent	No	ref	-	-
	Yes	0.45	0.18-1.16	0.098
History of severe chronic obstructive pulmonary disease	No	ref	-	-
	Yes	1.37	1.20-1.57	<0.001
Ascites <30 days prior to surgery	No	ref	-	-
	Yes	4.90	3.72-6.44	<0.001
Congestive heart failure <30 days prior to surgery	No	ref	-	-
	Yes	1.86	1.46-2.36	<0.001
Hypertension requiring medication	No	ref	-	-
	Yes	1.16	1.06-1.28	0.001
Disseminated cancer	No	ref	-	-
	Yes	2.41	2.17-2.67	<0.001
Immunosuppressant used for a chronic condition	No	ref	-	-
	Yes	1.35	1.16-1.57	<0.001
>10% weight loss in the 6 months prior to surgery	No	ref	-	-
	Yes	1.84	1.61-2.10	<0.001
Bleeding disorders	No	ref	-	-
	Yes	1.33	1.13-1.56	0.001
RBC transfusion <72 hours prior to surgery	No	ref	-	-
	Yes	1.75	1.27-2.42	0.001
Pre-operative blood urea nitrogen value		1.01	1.00-1.01	<0.001
Pre-operative creatinine value		1.04	0.95-1.14	0.415
Wound classification	1 - Clean	ref	-	-
	2 - Clean/Contaminated	0.95	0.84-1.07	0.390
	3 - Contaminated	1.09	0.93-1.29	0.270
	4 - Dirty/Infected	0.89	0.72-1.10	0.271
ASA classification	1 - No disturb	ref	-	-
	2 - Mild disturb	1.47	0.61-3.56	0.396
	3 - Severe disturb	3.08	1.28-7.45	0.012
	4 - Life threat	6.24	2.57-15.16	<0.001
	5 - Moribund	4.75	0.69-32.79	0.114
	None assigned	2.69	0.63-11.39	0.180
Wound closure	All layers fully closed	ref	-	-
	No layers surgically closed	4.17	3.13-5.55	<0.001
	Only deep layers closed	1.67	1.24-2.25	0.001
Sepsis within 48 hours prior to surgery	None	ref	-	-
	Sepsis	2.60	1.84-3.68	<0.001
	Septic shock	1.84	0.82-4.12	0.138
	SIRS	2.56	2.02-3.23	<0.001
Sepsis present at the time of surgery	No	ref	-	-
	Yes	0.77	0.52-1.15	0.206
Septic shock present at the time of surgery	No	ref	-	-
	Yes	4.79	2.92-7.83	<0.001
Deep incisional SSI present at the time of surgery	No	ref	-	-
	Yes	1.19	0.54-2.62	0.668
Organ/Space SSI present at the time of surgery	No	ref	-	-
	Yes	1.49	1.06-2.09	0.023
Pneumonia present at the time of surgery	No	ref	-	-
	Yes	3.52	2.23-5.57	<0.001
On ventilator > 48 hours present at the time of surgery	No	ref	-	-
	Yes	8.99	4.32-18.72	<0.001
Work relative value unit		1.01	1.01-1.02	<0.001
Total operation time		1.00	1.00-1.00	<0.001

Appendix 3

**Table 6:** NSQIP 2015-2019 univariable analysis of readmission BMI: body mass index; RBC: red blood cell; OD: odds ratio; MAC: monitored anesthesia care; IV: intravenous; CI: confidence interval; ASA: American Society of Anesthesiologists; SSI: surgical site infection; SIRS: systemic inflammatory response syndrome; NSQIP: National Surgical Quality Improvement Program

Characteristic		OR	95% CI	p-value
BMI category	18.5-25 kg/m^2^	Ref		
	<18.5 kg/m^2^	0.56	0.52-0.61	<0.001
	25-29.9 kg/m^2^	0.83	0.78-0.89	<0.001
	30-39.9 kg/m^2^	0.78	0.73-0.84	<0.001
	40-49.9 kg/m^2^	0.75	0.71-0.81	<0.001
	>50 kg/m^2^	0.58	0.55-0.63	<0.001
BMI		0.99	0.99-0.99	<0.001
Sex	Female	0.10	0.10-0.10	<0.001
	Male	1.15	1.12-1.17	<0.001
Race	American Indian or Alaska Native	0.13	0.11-0.15	<0.001
	Asian	0.79	0.79-0.91	0.002
	Black or African American	0.87	0.87-1.01	0.063
	Native Hawaiian or Pacific Islander	0.78	0.78-1.00	0.052
	Unknown/Not Reported	0.75	0.75-0.87	<0.001
	White	0.88	0.88-1.01	0.069
Hispanic ethnicity	No	0.11	0.11-0.11	<0.001
	Unknown	0.84	0.82-0.88	<0.001
	Yes	0.93	0.90-0.97	<0.001
Age		1.01	1.00-1.01	<0.001
Diabetes mellitus requiring therapy	Insulin	0.15	0.14-0.15	<0.001
	No	0.70	0.68-0.73	<0.001
	Non-insulin	0.75	0.72-0.79	<0.001
Current smoker within one year	No	0.10	0.10-0.11	<0.001
	Yes	1.24	1.21-1.27	<0.001
Dyspnea	At rest	0.17	0.15-0.20	<0.001
	Moderate exertion	0.74	0.64-0.86	<0.001
	No	0.62	0.54-0.72	<0.001
Functional health status	Independent	0.11	0.11-0.11	<0.001
	Partially dependent	1.62	1.50-1.75	<0.001
	Totally dependent	1.65	1.38-1.98	<0.001
Ventilator dependent	No	0.11	0.11-0.11	<0.001
	Yes	1.59	0.99-2.55	0.056
History of severe chronic obstructive pulmonary disease	No	0.11	0.11-0.11	<0.001
	Yes	1.52	1.46-1.59	<0.001
Ascites <30 days prior to surgery	No	0.11	0.11-0.11	<0.001
	Yes	2.16	1.85-2.51	<0.001
Congestive heart failure <30 days prior to surgery	No	0.11	0.11-0.11	<0.001
	Yes	1.65	1.47-1.84	<0.001
Hypertension requiring medication	No	0.10	0.10-0.10	<0.001
	Yes	1.15	1.13-1.17	<0.001
Disseminated cancer	No	0.11	0.10-0.11	<0.001
	Yes	1.43	1.38-1.48	<0.001
Immunosuppressant used for a chronic condition	No	0.11	0.10-0.11	<0.001
	Yes	1.48	1.43-1.54	<0.001
>10% weight loss in the 6 months prior to surgery	No	0.11	0.11-0.11	<0.001
	Yes	1.56	1.49-1.64	<0.001
Bleeding disorders	No	0.11	0.11-0.11	<0.001
	Yes	1.50	1.42-1.59	<0.001
RBC transfusion <72 hours prior to surgery	No	0.11	0.11-0.11	<0.001
	Yes	1.33	1.13-1.56	<0.001
Pre-operative blood urea nitrogen value		1.00	1.00-1.01	<0.001
Pre-operative creatinine value		1.12	1.10-1.14	<0.001
Wound classification	1 - Clean	0.09	0.09-0.09	<0.001
	2 - Clean/Contaminated	1.19	1.16-1.23	<0.001
	3 - Contaminated	1.56	1.50-1.63	<0.001
	4 - Dirty/Infected	1.54	1.46-1.62	<0.001
ASA classification	1 - No disturb	0.07	0.06-0.08	<0.001
	2 - Mild disturb	1.25	1.14-1.38	<0.001
	3 - Severe disturb	1.71	1.55-1.88	<0.001
	4 - Life threat	2.18	1.96-2.42	<0.001
	5 - Moribund	0.78	1.89-3.27	0.739
	None assigned	1.41	1.02-1.95	0.038
Wound closure	All layers fully closed	0.11	0.11-0.11	<0.001
	No layers surgically closed	1.09	0.94-1.26	0.255
	Only deep layers closed	1.25	1.14-1.37	<0.001
Sepsis within 48 hours prior to surgery	None	0.11	0.11-0.11	<0.001
	Sepsis	1.29	1.12-1.49	<0.001
	Septic shock	1.35	0.75-2.41	0.314
	SIRS	1.30	1.18-1.42	<0.001
Sepsis present at the time of surgery	No	0.04	0.04-0.04	<0.001
	Yes	2.83	2.48-3.22	<0.001
Septic shock present at the time of surgery	No	0.11	0.11-0.11	<0.001
	Yes	2.14	1.52-3.02	<0.001
Deep incisional SSI present at the time of surgery	No	0.11	0.11-0.11	<0.001
	Yes	5.24	4.29-6.41	<0.001
Organ/Space SSI present at the time of surgery	No	0.11	0.11-0.11	<0.001
	Yes	6.09	5.62-6.60	<0.001
Pneumonia present at the time of surgery	No	0.11	0.11-0.11	<0.001
	Yes	2.75	2.13-3.56	<0.001
On ventilator > 48 hours present at the time of surgery	No	0.11	0.11-0.11	<0.001
	Yes	1.53	0.85-2.76	0.154
Principal anesthesia technique	Epidural	0.13	0.09-0.17	<0.001
	General	0.86	0.64-1.16	0.323
	Local	0.72	0.25-2.09	0.545
	MAC/IV sedation	1.01	0.69-1.47	0.977
	None	0.77	0.35-1.69	0.519
	Other	1.07	0.69-1.66	0.753
	Regional	0.89	0.52-1.53	0.684
	Spinal	0.63	0.41-0.95	0.026
	Unknown	0.22	0.03-1.63	0.138
Quarter of admission	1	0.11	0.11-0.11	<0.001
	2	1.02	0.99-1.05	0.102
	3	1.04	1.01-1.07	0.004
	4	0.98	0.95-1.01	0.200
Work relative value unit		1.02	1.02-1.02	0.016
Total operation time		1.00	1.00-1.00	0.002

Appendix 4

**Table 7:** NSQIP 2015-2019 multivariable analysis of readmission BMI: body mass index; RBC: red blood cell; OD: odds ratio; MAC: monitored anesthesia care; IV: intravenous; CI: confidence interval; ASA: American Society of Anesthesiologists; SSI: surgical site infection; SIRS: systemic inflammatory response syndrome; NSQIP: National Surgical Quality Improvement Program

Characteristic		OR	95% CI	p-value
BMI category	18.5-25 kg/m^2^	ref	-	-
	<18.5 kg/m^2^	1.12	1.04-1.20	0.002
	25-29.9 kg/m^2^	0.94	0.91-0.97	<0.001
	30-39.9 kg/m^2^	0.91	0.88-0.94	<0.001
	40-49.9 kg/m^2^	0.76	0.73-0.79	<0.001
	>50 kg/m^2^	0.72	0.68-0.77	<0.001
Sex	Female	Ref	-	-
	Male	0.98	0.96-1.00	0.118
Race	American Indian or Alaska Native	ref	-	-
	Asian	0.83	0.71-0.97	0.017
	Black or African American	0.95	0.82-1.10	0.479
	Native Hawaiian or Pacific Islander	0.88	0.69-1.12	0.299
	Unknown/Not Reported	0.86	0.74-1.00	0.046
	White	0.94	0.81-1.08	0.377
Hispanic ethnicity	No	ref	-	-
	Unknown	0.98	0.93-1.03	0.455
	Yes	1.03	0.98-1.07	0.212
Age		1.00	1.00-1.00	<0.001
Diabetes mellitus requiring therapy	Insulin	ref	-	-
	No	0.80	0.77-0.83	<0.001
	Non-insulin	0.84	0.80-0.88	<0.001
Current smoker within one year	No	ref	-	-
	Yes	1.15	1.12-1.18	<0.001
Dyspnea	At rest	ref	-	-
	Moderate exertion	0.89	0.77-1.05	0.161
	No	0.82	0.71-0.96	0.012
Functional health status	Independent	ref	-	-
	Partially dependent	1.41	1.30-1.53	<0.001
	Totally dependent	1.55	1.29-1.87	<0.001
Ventilator dependent	No	ref	-	-
	Yes	1.11	0.64-1.90	0.714
History of severe chronic obstructive pulmonary disease	No	ref	-	-
	Yes	1.27	1.21-1.33	<0.001
Ascites <30 days prior to surgery	No	ref	-	-
	Yes	1.69	1.45-1.98	<0.001
Congestive heart failure <30 days prior to surgery	No	ref	-	-
	Yes	1.26	1.12-1.42	<0.001
Hypertension requiring medication	No	ref	-	-
	Yes	1.08	1.06-1.11	<0.001
Disseminated cancer	No	ref	-	-
	Yes	1.22	1.18-1.27	<0.001
Immunosuppressant used for a chronic condition	No	ref	-	-
	Yes	1.40	1.35-1.45	<0.001
>10% weight loss in the 6 months prior to surgery	No	ref	-	-
	Yes	1.12	1.07-1.18	<0.001
Bleeding disorders	No	ref	-	-
	Yes	1.29	1.22-1.37	<0.001
RBC transfusion <72 hours prior to surgery	No	ref	-	-
	Yes	1.01	0.86-1.19	0.927
Pre-operative blood urea nitrogen value		1.00	1.00-1.00	0.921
Pre-operative creatinine value		1.08	1.06-1.11	<0.001
Wound classification	1 - Clean	ref	-	-
	2 - Clean/Contaminated	1.12	1.08-1.15	<0.001
	3 - Contaminated	1.30	1.25-1.36	<0.001
	4 - Dirty/Infected	1.05	0.99-1.11	0.098
ASA classification	1 - No disturb	ref	-	-
	2 - Mild disturb	1.15	1.04-1.27	0.005
	3 - Severe disturb	1.43	1.29-1.57	<0.001
	4 - Life threat	1.58	1.41-1.76	<0.001
	5 - Moribund	0.50	0.12-2.16	0.356
	None assigned	1.30	0.93-1.80	0.120
Wound closure	All layers fully closed	ref	-	-
	No layers surgically closed	0.95	0.82-1.11	0.532
	Only deep layers closed	1.10	1.00-1.21	0.042
Sepsis within 48 hours prior to surgery	None	ref	-	-
	Sepsis	0.93	0.80-1.09	0.389
	Septic shock	0.81	0.42-1.56	0.535
	SIRS	1.18	1.07-1.30	0.001
Sepsis present at the time of surgery	No	ref	-	-
	Yes	1.04	0.92-1.17	0.558
Septic shock present at the time of surgery	No	ref	-	-
	Yes	0.83	0.56-1.22	0.350
Deep incisional SSI present at the time of surgery	No	ref	-	-
	Yes	4.91	3.98-6.06	<0.001
Organ/Space SSI present at the time of surgery	No	ref	-	-
	Yes	5.00	4.58-5.46	<0.001
Pneumonia present at the time of surgery	No	ref	-	-
	Yes	1.97	1.51-2.58	<0.001
On ventilator > 48 hours present at the time of surgery	No	ref	-	-
	Yes	0.97	0.50-1.87	0.919
Quarter of admission	1	ref	-	-
	2	1.03	1.00-1.06	0.077
	3	1.03	1.00-1.06	0.036
	4	0.98	0.95-1.01	0.210
Work relative value unit		1.00	1.00-1.00	<0.001
Total operation time		1.00	1.00-1.00	<0.001

Appendix 5

**Table 8:** NSQIP 2015-2019 univariable analysis of reoperation BMI: body mass index; RBC: red blood cell; OD: odds ratio; MAC: monitored anesthesia care; IV: intravenous; CI: confidence interval; ASA: American Society of Anesthesiologists; SSI: surgical site infection; SIRS: systemic inflammatory response syndrome; NSQIP: National Surgical Quality Improvement Program

Characteristic		OR	95% CI	p-value
BMI category	18.5-25 kg/m^2^	REF		
	<18.5 kg/m^2^	1.32	1.20-1.45	<0.001
	25-29.9 kg/m^2^	0.88	0.84-0.92	<0.001
	30-39.9 kg/m^2^	0.89	0.85-0.92	<0.001
	40-49.9 kg/m^2^	0.74	0.70-0.78	<0.001
	>50 kg/m^2^	0.65	0.59-0.71	<0.001
BMI		1.01	0.99-0.99	<0.001
Sex	Female	0.04	0.04-0.04	<0.001
	Male	1.27	1.23-1.31	<0.001
Race	American Indian or Alaska Native	0.06	0.05-0.07	<0.001
	Asian	0.70	0.56-0.86	0.001
	Black or African American	0.83	0.68-1.01	0.063
	Native Hawaiian or Pacific Islander	0.72	0.50-1.03	0.068
	Unknown/Not Reported	0.73	0.60-0.90	0.002
	White	0.81	0.66-0.98	0.032
Hispanic ethnicity	No	0.05	0.04-0.05	<0.001
	Unknown	0.92	0.87-0.97	0.001
	Yes	0.93	0.88-0.99	0.020
Age		1.01	1.00-1.01	<0.001
Diabetes mellitus requiring therapy	Insulin	0.05	0.05-0.05	<0.001
	No	0.91	0.86-0.97	0.002
	Non-insulin	0.86	0.80-0.92	<0.001
Current smoker within one year	No	0.04	0.04-0.04	<0.001
	Yes	1.45	1.40-1.51	<0.001
Dyspnea	At rest	0.07	0.06-0.08	<0.001
	Moderate exertion	0.78	0.62-0.96	0.022
	No	0.64	0.52-0.80	<0.001
Functional health status	Independent	0.04	0.04-0.04	<0.001
	Partially dependent	1.68	1.51-1.88	<0.001
	Totally dependent	2.40	1.93-2.99	<0.001
Ventilator dependent	No	0.04	0.04-0.05	<0.001
	Yes	4.80	3.09-7.46	<0.001
History of severe chronic obstructive pulmonary disease	No	0.04	0.04-0.04	<0.001
	Yes	1.70	1.60-1.81	<0.001
Ascites <30 days prior to surgery	No	0.04	0.04-0.05	<0.001
	Yes	1.57	1.23-1.99	<0.001
Congestive heart failure <30 days prior to surgery	No	0.04	0.04-0.05	<0.001
	Yes	1.73	1.48-2.02	<0.001
Hypertension requiring medication	No	0.04	0.04-0.04	<0.001
	Yes	1.11	1.08-1.14	<0.001
Disseminated cancer	No	0.04	0.04-0.04	<0.001
	Yes	1.12	1.06-1.19	<0.001
Immunosuppressant used for a chronic condition	No	0.04	0.04-0.04	<0.001
	Yes	1.24	1.17-1.31	<0.001
>10% weight loss in the 6 months prior to surgery	No	0.04	0.04-0.04	<0.001
	Yes	1.55	1.45-1.66	<0.001
Bleeding disorders	No	0.04	0.04-0.04	<0.001
	Yes	1.47	1.36-1.59	<0.001
RBC transfusion <72 hours prior to surgery	No	0.04	0.04-0.05	<0.001
	Yes	1.68	1.37-2.07	<0.001
Pre-operative blood urea nitrogen value		1.00	1.00-1.01	<0.001
Pre-operative creatinine value		1.10	1.07-1.14	<0.001
Wound classification	1 - Clean	0.05	0.05-0.05	<0.001
	2 - Clean/Contaminated	0.75	0.72-0.78	<0.001
	3 - Contaminated	1.07	1.01-1.13	0.020
	4 - Dirty/Infected	1.21	1.13-1.29	<0.001
ASA classification	1 - No disturb	0.04	0.03-0.04	<0.001
	2 - Mild disturb	1.09	0.96-1.24	0.204
	3 - Severe disturb	1.32	1.16-1.50	<0.001
	4 - Life threat	1.81	1.57-2.09	<0.001
	5 - Moribund	2.39	0.73-7.81	0.151
	None assigned	1.07	0.66-1.75	0.778
Wound closure	All layers fully closed	0.04	0.04-0.04	<0.001
	No layers surgically closed	2.56	2.20-2.97	<0.001
	Only deep layers closed	1.67	1.49-1.88	<0.001
Sepsis within 48 hours prior to surgery	None	0.04	0.04-0.04	<0.001
	Sepsis	1.83	1.54-2.18	<0.001
	Septic shock	3.59	2.04-6.31	<0.001
	SIRS	1.45	1.27-1.64	<0.001
Sepsis present at the time of surgery	No	0.04	0.04-0.04	0.04
	Yes	2.83	2.48-3.22	2.83
Septic shock present at the time of surgery	No	0.04	0.04-0.05	0.04
	Yes	9.07	6.73-12.22	9.07
Deep incisional SSI present at the time of surgery	No	0.04	0.04-0.05	0.04
	Yes	5.21	4.07-6.67	5.21
Organ/Space SSI present at the time of surgery	No	0.04	0.04-0.04	0.04
	Yes	5.58	5.06-6.16	5.58
Pneumonia present at the time of surgery	No	0.04	0.04-0.05	0.04
	Yes	2.70	1.90-3.83	2.70
On ventilator > 48 hours present at the time of surgery	No	0.04	0.04-0.05	0.04
	Yes	11.58	7.51-17.88	11.58
Principal anesthesia technique	Epidural	0.06	0.04-0.09	<0.001
	General	0.77	0.51-1.16	0.209
	Local	0.00	0.00-0.00	0.836
	MAC/IV sedation	1.22	0.73-2.03	0.442
	None	1.68	0.73-3.87	0.224
	Other	0.56	0.27-1.15	0.113
	Regional	1.25	0.63-2.47	0.523
	Spinal	0.56	0.31-1.01	0.056
	Unknown	0.00	0.00-0.00	0.856
Quarter of admission	1	0.05	0.04-0.05	<0.001
	2	0.95	0.91-0.99	0.024
	3	0.96	0.92-1.00	0.040
	4	0.96	0.92-1.00	0.035
Work relative value unit		1.00	1.00-1.01	<0.001
Total operation time		1.00	1.00-1.00	<0.001

Appendix 6

**Table 9:** NSQIP 2015-2019 multivariable analysis of reoperation BMI: body mass index; RBC: red blood cell; OD: odds ratio; MAC: monitored anesthesia care; IV: intravenous; CI: confidence interval; ASA: American Society of Anesthesiologists; SSI: surgical site infection; SIRS: systemic inflammatory response syndrome; NSQIP: National Surgical Quality Improvement Program

Characteristic		OR	95% CI	p-value
BMI category	18.5-25 kg/m^2^	ref	-	-
	<18.5 kg/m^2^	1.23	1.12-1.36	<0.001
	25-29.9 kg/m^2^	0.87	0.83-0.90	<0.001
	30-39.9 kg/m^2^	0.88	0.84-0.92	<0.001
	40-49.9 kg/m^2^	0.85	0.80-0.91	<0.001
	>50 kg/m^2^	0.78	0.70-0.85	<0.001
Sex	Female	ref	-	-
	Male	1.16	1.12-1.20	<0.001
Race	American Indian or Alaska Native	ref	-	-
	Asian	0.74	0.60-0.93	0.009
	Black or African American	0.91	0.74-1.12	0.365
	Native Hawaiian or Pacific Islander	0.81	0.57-1.17	0.263
	Unknown/Not Reported	0.82	0.67-1.02	0.073
	White	0.87	0.71-1.06	0.156
Hispanic ethnicity	No	ref	-	-
	Unknown	1.02	0.95-1.10	0.553
	Yes	1.03	0.97-1.10	0.302
Age		1.00	1.00-1.00	0.005
Diabetes mellitus requiring therapy	Insulin	ref	-	-
	No	1.06	1.00-1.12	0.067
	Non-insulin	0.97	0.90-1.04	0.365
Current smoker within one year	No	ref	-	-
	Yes	1.32	1.27-1.37	<0.001
Dyspnea	At rest	ref	-	-
	Moderate exertion	1.01	0.81-1.27	0.880
	No	0.93	0.75-1.17	0.555
Functional health status	Independent	ref	-	-
	Partially dependent	1.36	1.22-1.52	<0.001
	Totally dependent	1.93	1.54-2.42	<0.001
Ventilator dependent	No	ref	-	-
	Yes	1.25	0.69-2.26	0.470
History of severe chronic obstructive pulmonary disease	No	ref	-	-
	Yes	1.35	1.27-1.44	<0.001
Ascites <30 days prior to surgery	No	ref	-	-
	Yes	1.18	0.92-1.49	0.208
Congestive heart failure <30 days prior to surgery	No	ref	-	-
	Yes	1.27	1.08-1.49	0.004
Hypertension requiring medication	No	ref	-	-
	Yes	1.07	1.03-1.10	<0.001
Disseminated cancer	No	ref	-	-
	Yes	1.01	0.95-1.07	0.849
Immunosuppressant used for a chronic condition	No	ref	-	-
	Yes	1.18	1.11-1.25	<0.001
>10% weight loss in the 6 months prior to surgery	No	ref	-	-
	Yes	1.19	1.11-1.27	<0.001
Bleeding disorders	No	ref	-	-
	Yes	1.22	1.12-1.32	<0.001
RBC transfusion <72 hours prior to surgery	No	ref	-	-
	Yes	1.18	0.95-1.47	0.133
Pre-operative blood urea nitrogen value		1.00	1.00-1.00	0.441
Pre-operative creatinine value		1.03	0.99-1.07	0.109
Wound classification	1 - Clean	ref	-	-
	2 - Clean/Contaminated	0.75	0.72-0.78	<0.001
	3 - Contaminated	0.92	0.87-0.97	0.005
	4 - Dirty/Infected	0.76	0.70-0.82	<0.001
ASA classification	1 - No disturb	ref	-	-
	2 - Mild disturb	1.01	0.89-1.15	0.865
	3 - Severe disturb	1.12	0.98-1.28	0.100
	4 - Life threat	1.26	1.09-1.47	0.002
	5 - Moribund	1.30	0.35-4.88	0.697
	None assigned	0.91	0.56-1.49	0.706
Wound closure	All layers fully closed	ref	-	-
	No layers surgically closed	1.99	1.70-2.34	<0.001
	Only deep layers closed	1.42	1.26-1.60	<0.001
Sepsis within 48 hours prior to surgery	None	ref	-	-
	Sepsis	0.90	0.74-1.10	0.299
	Septic shock	0.52	0.25-1.07	0.077
	SIRS	1.25	1.10-1.43	0.001
Sepsis present at the time of surgery	No	ref	-	-
	Yes	1.45	1.25-1.69	<0.001
Septic shock present at the time of surgery	No	ref	-	-
	Yes	3.58	2.52-5.08	<0.001
Deep incisional SSI present at the time of surgery	No	ref	-	-
	Yes	3.63	2.80-4.70	<0.001
Organ/Space SSI present at the time of surgery	No	ref	-	-
	Yes	4.06	3.64-4.54	<0.001
Pneumonia present at the time of surgery	No	ref	-	-
	Yes	1.44	0.99-2.10	0.056
On ventilator > 48 hours present at the time of surgery	No	ref	-	-
	Yes	4.60	2.64-8.02	<0.001
Quarter of admission	1	ref	-	-
	2	0.96	0.92-1.00	0.049
	3	0.95	0.91-0.99	0.012
	4	0.96	0.92-1.00	0.041
Work relative value unit		0.99	0.99-0.99	<0.001
Total operation time		1.00	1.00-1.00	<0.001

Appendix 7

**Table 10:** Mean square error and C-statistics for multivariable models

Multivariable models	Mean square error	C-statistic
Acute renal failure	0.008	0.78
Mortality	0.006	0.83
Readmission	0.086	0.63
Reoperation	0.040	0.63
